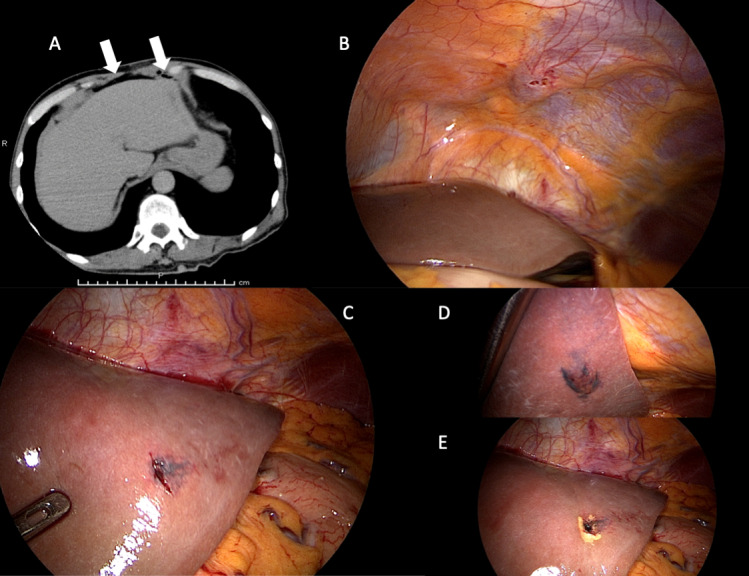# Self-inflicted very-low-velocity penetrating head injury: A CARE-compliant case report

**DOI:** 10.1097/MD.0000000000037896

**Published:** 2024-05-03

**Authors:** Koshi Ota, Hitoshi Kobata, Shunsuke Tomonishi, Kanna Ota, Akira Takasu

**Affiliations:** a Department of Emergency and Critical Care Medicine, Osaka Medical and Pharmaceutical University, Takatsuki City, Osaka, Japan.

**Keywords:** computed tomography, magnetic resonance imaging, penetrating head injury (PHI), traumatic brain injury

## Abstract

**Rationale:**

Low-velocity penetrating head injury (PHI) is rare, comprising 0.2% to 0.4% of head traumas, but can be devastating and is associated with significant morbidity and mortality. No previous case of very-low-velocity PHI due to self-inflicted stabbing with a gimlet has been reported.

**Patient concerns:**

A 62-year-old man was admitted to the hospital with bleeding head and abdominal wounds after stabbing his abdomen with a gimlet, and then hammering the same gimlet into his forehead and removing the gimlet himself.

**Diagnoses:**

Upon examination at admission, stab wounds were present on the forehead and the right upper quadrant. Computed tomography (CT) of the head revealed a bone defect in the left frontal bone and showed the intracranial path of the gimlet surrounded by mild hemorrhage and pneumocephalus. Magnetic resonance imaging (MRI) confirmed a small amount of hemorrhage with pneumocephalus but no vascular injury.

**Interventions:**

Conservative treatment without surgery.

**Outcomes:**

Follow-up MRI on hospital day 58 showed no abscess or traumatic intracranial aneurysm. The patient achieved full recovery of motor and mental functions with conservative treatment and was discharged on hospital day 69.

**Lessons:**

Very-low-velocity PHI might be successfully treated with conservative treatment.

## 1. Introduction

Penetrating head injury (PHI) is relatively rare in Japan.^[1]^ Two major classes of projectiles are implicated in PHI, missiles and nonmissiles, which are defined by their velocity on impact. Nonmissiles are categorized as low-velocity PHI (<100 m/s) and cause tissue damage by local laceration and maceration, whereas missiles cause injury via kinetic waves and thermal energy.^[2]^ Low-velocity PHI is rare, comprising 0.2%–0.4% of head trauma injuries, but can be devastating and is associated with significant morbidity and mortality.^[2,3]^ Intracranial penetration has been reported for various types of low-velocity foreign bodies, including nails from a nail gun, chopsticks, an umbrella tip, eyeglass earpiece, toilet brush handle, fern, toothbrush, door key, fork, metal shelving bar, knitting needle, and tree branches.^[1,4]^ In one-quarter of adult cases of PHI, intracranial penetration is via the orbit.^[2]^ To the best of our knowledge, no previous case of very low-velocity PHI due to self-inflicted stabbing with a gimlet has been reported. In the most similar case reported in Japan, PHI was caused by an icepick. The patient in that case fell from the fifth floor with the icepick already partially penetrating the skull, followed by complete penetration of the skull at a much higher velocity upon impact with the ground.^[5]^ In the present case, very low-velocity PHI occurred by hammering the gimlet through the skull.

The major risks associated with low-velocity PHI are traumatic cerebral aneurysm, hemorrhage, and infection.^[1,2,6]^ No complications associated with very-low-velocity PHI have been reported.

Here, we describe a case of very low-velocity PHI caused by self-inflicted stabbing with a gimlet that was successfully treated non-surgically and without complications.

## 2. Case presentation

A 62-year-old man was transported to our hospital with several bleeding wounds. He had stabbed his abdomen with a gimlet, stabbed his forehead with the same gimlet using a hammer, and then removed the gimlet by himself. He had also cut his forearm with a cutter knife. His wife had found the patient lying on a bed. He told her that he had intended to commit suicide by self-inflicted injury because he was suffering chronic pain due to rectal cancer resection. The patient had a medical history of diabetes mellitus and rectal cancer resection at the age of 55 years. He had been prescribed linagliptin 5 mg once daily; metformin hydrochloride 250 mg, celecoxib 100 mg, rebamipide 100 mg, and mirogabalin besilate 2.5 mg twice daily; flunitrazepam 2 mg and trazodone 50 mg once daily before bed; and acetaminophen 500 mg 3 times daily. His habitual activities and family history were unremarkable. He was a retired dump truck driver and lived with his wife and daughter. He was a previous smoker (30 pack-years) but had stopped at the age of 53 years, and did not drink alcohol. On arrival at the emergency room, his vital signs were as follows: temperature, 35.4°C; heart rate, 90 beats/min with regular rhythm; respiratory rate, 24 breaths/min; blood pressure, 111/79 mm Hg; and oxygen saturation, 98% on room air. Glasgow Coma Scale score on arrival was 13 (E3V4M6), indicating altered consciousness due to brain injury.

## 3. Outcomes

**Figure 1.**
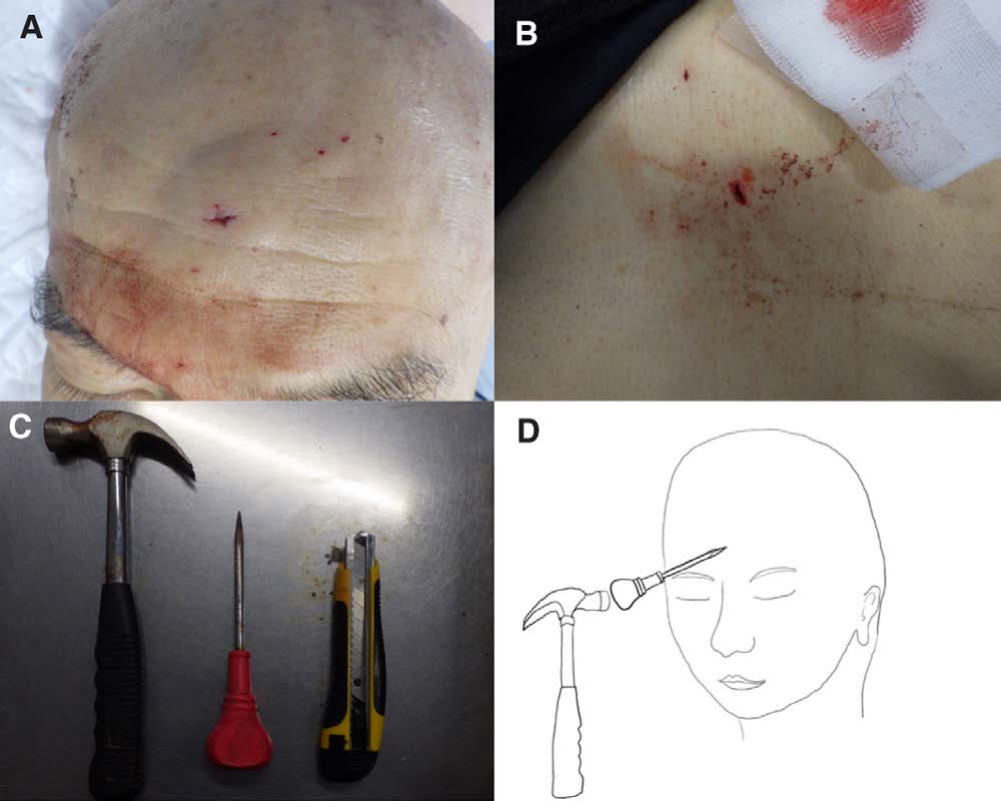
Photographs show locations of the stab wounds in the center of the forehead (A) and in the right upper quadrant of the abdomen (B) (left upper corner, cranial; right lower corner, caudal). The hammer, gimlet, and cutter knife (C) are shown (from left to right). The diagram (D) shows the position and angle of stabbing with the gimlet.

Upon examination, a stab wound was found on the forehead (Fig. 1A). The trachea was central, and no crackles or decreased breath sounds were heard on auscultation. A stab wound was present in the right upper quadrant (Fig. 1B) of the abdomen, and there was no abdominal distension. The cranial nerves were normal and no obvious neurological abnormalities were noted. Bruising was seen around the right eye. Pupil diameter was 3.0 mm on each side. Both eyes showed reactivity to light. Examination of the limbs showed no abnormalities other than a laceration on the left arm. Figure 1C shows the gimlet, cutter knife, and hammer used to inflict the injuries. Figure 1D shows the angle of penetration of the gimlet.

**Figure 2.**
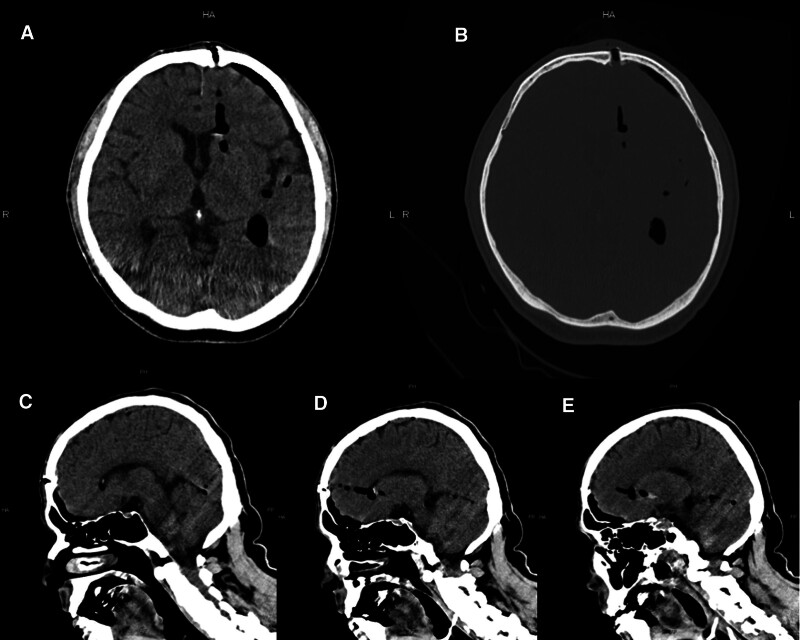
CT images of the head were obtained at the time of arrival at the hospital. Axial images demonstrate a frontal bone defect slightly to the left of the midline, and the route taken by the gimlet from the left frontal lobe through the anterior horn of the lateral ventricle, to the head of the caudate nucleus, putamen, and globus pallidus. A small amount of hemorrhage and air are present in the lateral ventricle and in the subarachnoid and subdural spaces (A and B). Sagittal reconstructions show the route of the gimlet through the frontal lobe, anterior horn of the lateral ventricle, head of the caudate nucleus, left putamen, and globus pallidus (C–E).

Computed tomography (CT) of the head showed the route of the gimlet, from the left frontal bone through the left frontal lobe to reach the anterior horn of the lateral ventricle and the head of the caudate nucleus, putamen, and globus pallidus. A small amount of hemorrhage and air was noted in the lateral ventricle and in the subarachnoid and subdural spaces (Fig. 2A–E). CT revealed free air in the abdomen (Supplemental Figure 1A, http://links.lww.com/MD/M321). Exploratory laparoscopy (Supplemental Figure 1B–E, http://links.lww.com/MD/M321) performed on the day of admission identified oozing from the left lobe of the liver, and cauterization was performed. Ceftriaxone 2.0 g was initiated for head injury and abdominal injury on the day of admission, which was increased to 2.0 g twice daily the following day with the addition of vancomycin.

**Figure 3.**
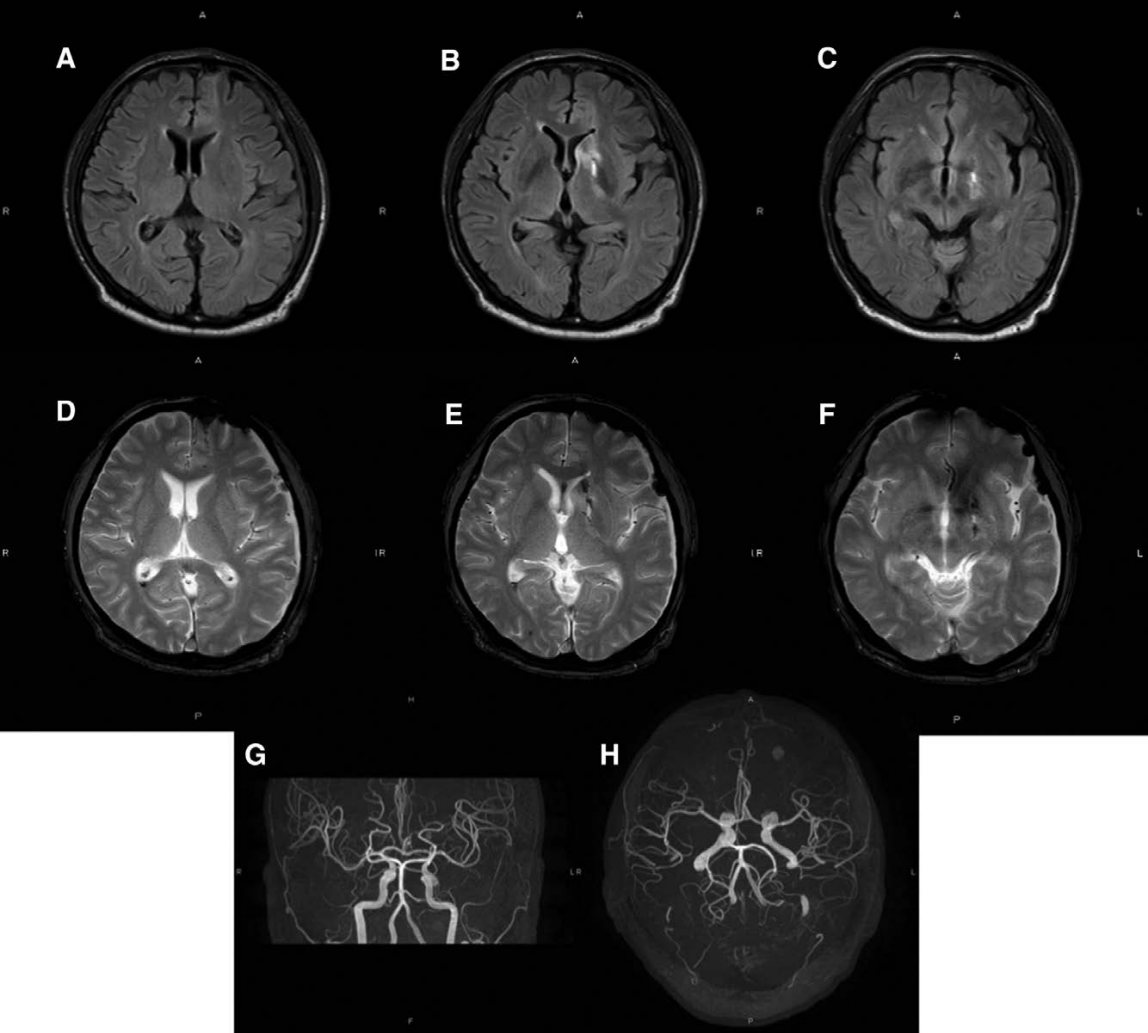
MR images of the brain. Axial FLAIR images acquired on hospital day 5 show no brain abscess or hemorrhagic enlargement (A–C). On axial T2* imaging, a small amount of hemorrhage is observed along the route taken by the gimlet (D–F). There is no evidence of traumatic cerebral aneurysm formation on magnetic resonance angiography (G and H).

**Figure 4.**
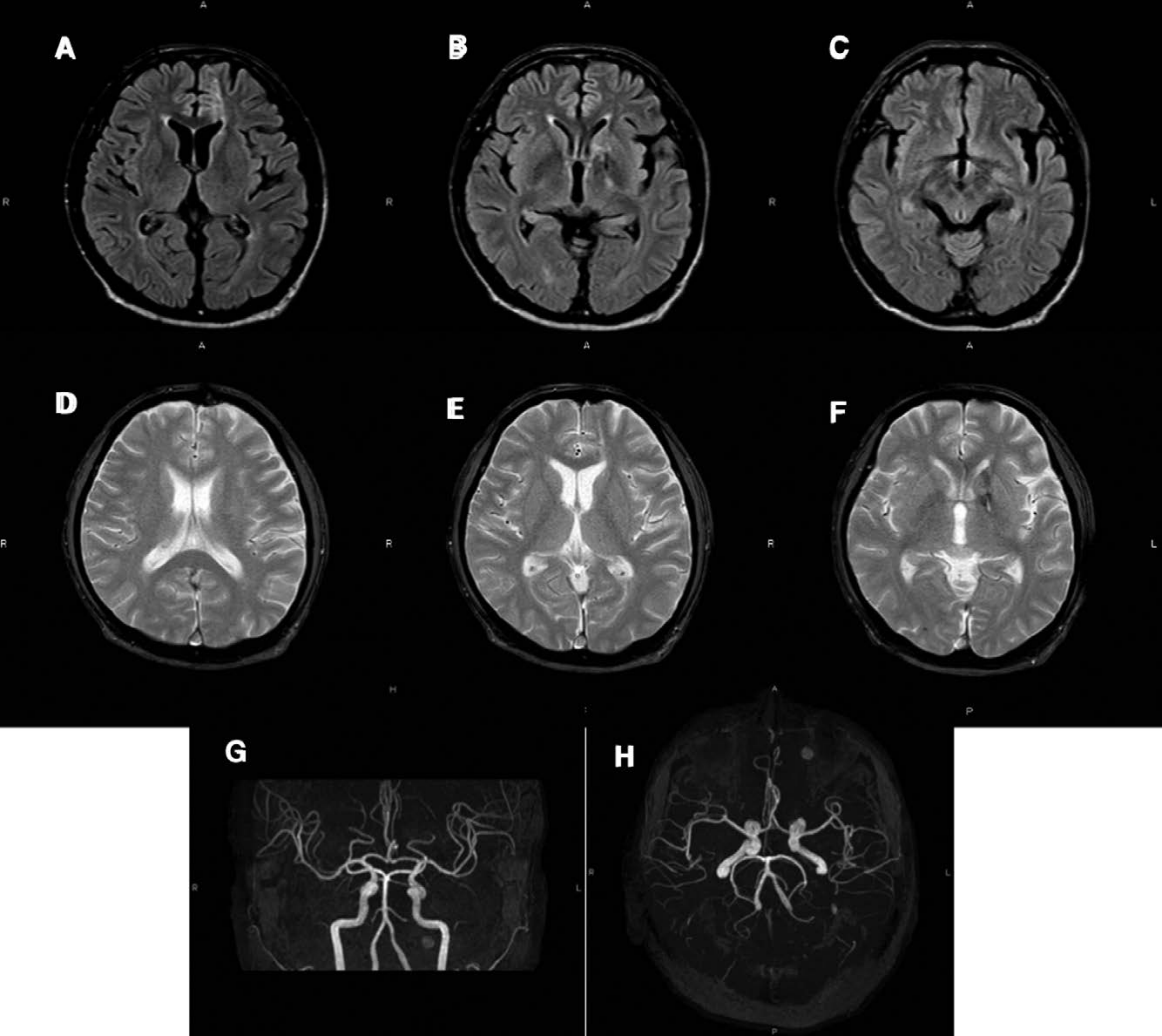
Axial FLAIR images acquired on hospital day 58 show no brain abscess or hemorrhagic enlargement (A–C). The amount of hemorrhage along the intracerebral route of the gimlet on axial T2* imaging is less than on hospital day 5 (D–F). There is no evidence of traumatic cerebral aneurysm formation (G and H).

Magnetic resonance imaging (MRI) of the head performed on hospital day 5 showed no evidence of brain abscess, brain contusion, hemorrhage enlargement, or traumatic cerebral aneurysm formation (Fig. 3A–H). The patient was transferred to the psychiatric ward on hospital day 8 without any complications. Follow-up MRI of the brain performed on hospital day 58 also found no evidence of brain abscess, hemorrhage enlargement, or traumatic aneurysm (Fig. 4A–H). He had no deterioration of cognitive function and his physical and mental functions had returned to the levels prior to the injuries. He complained of rectal pain, but the pain resolved from Numeric Rating Scale 10 to 5 with administration of mirogabalin besilate 10 mg twice daily and tramadol hydrochloride combined with acetaminophen 4 times daily, and he was discharged to home on hospital day 69.

## 4. Discussion

We encountered a case of very low-velocity PHI caused by self-inflicted stabbing with a gimlet that was successfully treated non-surgically and without complications. Low-velocity PHI can be devastating is associated with significant morbidity and mortality, and requires neurosurgical intervention with multidisciplinary treatments. In contrast to previous reports of low-velocity PHI, the present case was of very-low-velocity (<10 cm/s). Delayed infection was a major concern, but a follow-up MRI showed no evidence of infection.

Several mechanisms potentially explain how the patient recovered without suffering major impairment of brain function. First, kinetic energy upon impact is defined by velocity and mass (1/2 × mass × velocity^2^).^[7]^ In our case, the kinetic energy of the gimlet was very small because the velocity was so low, and thus the patient suffered very little damage to the brain. Second, the patient suffered no injury to vessels, which were easily pushed aside by the slow-moving gimlet. Accordingly, we observed no direct vessel injury in our case. Third, the patient removed the gimlet from his head by himself, which might have prevented the need for aggressive surgical intervention. If the gimlet had been retained in the brain, neurosurgical intervention would have been required and aggressive debridement might have been necessary. Objects penetrating the brain are typically removed prudently under fluoroscopic guidance during craniotomy. However, it is unsafe to remove a foreign body lodged in the brain in the prehospital setting without assessing vessels and damage to the brain. Fourth, antibiotics were initiated soon after admission to the hospital, which might have prevented brain abscesses and infection of the central nervous system. Fifth, as the gimlet was made of metal, no fragments were retained after removal, as can occur in the case of a wooden object such as a chopstick. Small fragments from foreign bodies that remain in place for years can cause brain abscesses or neurological symptoms such as meningitis or seizures.^[8–10]^ Finally, the path of the gimlet missed major intracranial vessels and sagittal sinuses. The patient placed the gimlet a little to the left of the center of the frontal bone rather than through the orbit, nasal cavities, oral cavity, or skull base.

Although there are numerous reports internationally of low-velocity PHI, we could not find any similar cases of very low-velocity PHI. Very-low-velocity PHI differs from low-velocity PHI in terms of its lower kinetic energy, and thus vessel injuries are rare, as mentioned above. Several PHIs with chopsticks have been reported, with velocity much higher than in the present case, and in most cases the PHI injury was transorbital.^[6,8,10]^ It might be possible to treat low-velocity PHI with antibiotic administration alone. However, the importance of CT and MRI should be emphasized. If vessel injury, traumatic cerebral aneurysm, hemorrhage, or infection are observed on CT or MRI, intensive surgical intervention should be performed without delay.

There is neither a previous case report nor guideline to treat very-low-velocity PHI like this case as potential constraints or challenges. We need to accumulate more similar cases to treat very low-velocity PHI patients safely and effectively.

## 5. Conclusion

We treated a patient who presented with very-low-velocity PHI caused by self-inflicted stabbing with a gimlet. Very-low-velocity PHI might be successfully treated with conservative treatment.

## Author contributions

**Conceptualization:** Koshi Ota.

**Data curation:** Shunsuke Tomonishi, Kanna Ota.

**Supervision:** Hitoshi Kobata, Akira Takasu.

**Writing – original draft:** Koshi Ota.

**Writing – review & editing:** Koshi Ota, Hitoshi Kobata, Shunsuke Tomonishi, Kanna Ota, Akira Takasu.

## Supplementary Material

**Figure:**